# Imaging and Biochemical Markers for Osteoarthritis

**DOI:** 10.3390/diagnostics11071205

**Published:** 2021-07-02

**Authors:** Benny Antony, Ambrish Singh

**Affiliations:** Menzies Institute for Medical Research, University of Tasmania, Hobart 7000, Australia; ambrishagastya@gmail.com

Osteoarthritis (OA) is the most common form of arthritis in adults that affects more than 500 million people globally [1]. The burden of OA is increasing due to the aging population and the rising prevalence of obesity or overweight in the general population globally [2,3]. The World Health Organization (WHO) has designated the 2020–2030 decade as the “Decade of Healthy Ageing,” which highlights the need to address chronic diseases with no cure, such as OA, that have a substantial impact on the health-related quality of life (HRQoL) [4,5].

There are no disease-modifying OA drugs (DMOADs) approved for the treatment of OA that can arrest, slow, or reverse the progression of structural damage of the joint [6]. Over the last decade, several therapeutic options have shown promising potential as a structure-modifying therapy in OA [6]. However, none has yet seen the light of the day to be designated as a DMOAD [7]. The reasons for the translational failure of DMOADs are likely manifold [8,9,10,11,12]; however, the main reasons for the failure are the lack of sensitive biomarkers that can substantiate the patient characterization, define disease progression, and ascertain the clinical benefit in improving disease progression [6].

The US Food and Drug Administration (FDA)–National Institutes of Health (NIH) defines biomarker as a defined characteristic that is measured as an indicator of normal biological processes, pathogenic processes, or biological responses to an exposure or intervention, including therapeutic interventions. Biomarkers may include molecular (proteins, protein fragments, metabolites, carbohydrates, genomic RNA and DNA biomarkers), histologic, radiographic, or physiologic characteristics [13].

Categories of biomarkers include:

- Susceptibility/risk biomarker;
- Diagnostic biomarker;
- Monitoring biomarker;
- Prognostic biomarker;
- Predictive biomarker;
- Pharmacodynamic/response biomarker;
- Safety biomarker.

## 1. Advancement in OA Biomarker Research

**Figure 1:**
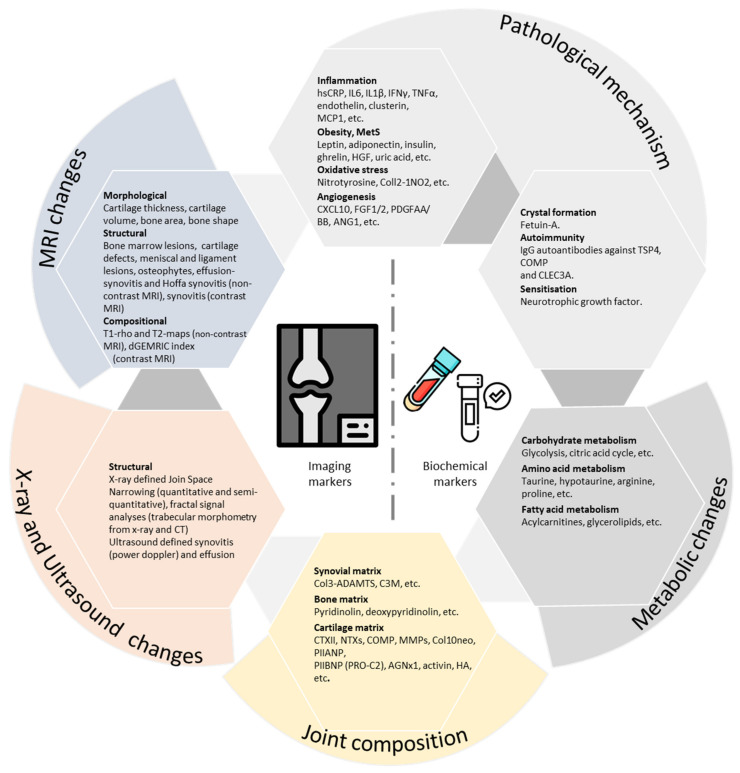
A non-exhaustive representation of osteoarthritis biomarkers. Note: Adapted from: van Spil WE, Szilagyi IA. Osteoarthritis year in review 2019: biomarkers (biochemical markers). Osteoarthritis and cartilage. 1 March 2020;28(3):296-315. Icon designed by Freepik from www.flaticon.com. Access date: 24 June 2021. AGNx1: ADAMTS-degraded aggrecan; ANG: angiopoietin; BML: bone marrow lesion; C3M: collagen type III degraded by matrix metalloproteinase; CLEC3A: C-type lectin domain family 3 member A; Coll2-1NO2: nitrated epitope of the a-helical region of type II collagen; COL3-ADAMTS: collagen type III cleavage product derived from ADAMTS; Col10neo: collagen typeX neo-epitope; COMP: cartilage oligomeric matrix protein; CTXII: C-terminal cross-linked telopeptide of collagen type II; CXCL10: C-X-C motif chemokine 10; dGEMRIC: delayed gadolinium-enhanced MRI of cartilage; FGF: fibroblast growth factor; HGF: hepatic growth factor, hsCRP: high sensitivity C-reactive protein; IL: interleukin; IFN: interferon; MCP: monocyte chemoattractant protein; MMPs: matrix metalloproteinases; NTX: N-telopeptide crosslinks; PDGF: platelet-derived growth factor; PIIANP, PIIBNP: N-terminal propeptide of type II collagen, splice variants IIA and IIB, respectively; TNF: tumour necrosis factor; TSP: thrombospondin; US: ultrasound; MRI: magnetic resonance imaging.

Over the last decade and a half, advances have been made in the evaluation and qualification of OA-specific biomarkers [14]. As a result, BIPEDS classification is now widely used to categorize these OA-specific biomarkers according to their utility based on the key parameters, which are: Burden of disease (B), Investigative (I), Prognostic (P), Efficacy of intervention (E), Diagnostic (D), and Safety (S) [14,15]. The Osteoarthritis Research Society International (OARSI)/US FDA initiative further classified the OA-specific biomarkers into two major groups: wet biomarkers and dry biomarkers [16]. The wet or soluble biomarkers represent a modulation of endogenous substances in body fluids and are measured in urine, blood, serum, plasma, or synovial fluid (Figure 1). The dry biomarkers include imaging markers from X-ray, ultrasound, and MRI [15,16] (Figure 1).

There have been tremendous advances in research on various biomarkers, including imaging, biochemical, and molecular biomarkers, as potential markers to predict the incidence and progression of OA [17]. The prominent scientific organizations, such as the NIH, through the Foundation for the National Institutes of Health (FNIH), consisting of a team of leading OA societies and clinicians, have invested millions of dollars in exploring radiographic measures, MRI measures, and biochemical markers of knee OA [18]. However, several of these markers are still exploratory. 

## 2. Imaging Biomarkers

Radiographically (X-ray) defined Joint Space Narrowing (JSN) is currently the only recommended imaging endpoint by both the US FDA and European Agency for the Evaluation of Medicinal Products (EMEA) guidance documents for clinical trials of DMOADs [19]. However, JSN has limited sensitivity to assess structural changes as the joint space is constituted by cartilage and meniscal integrity. Moreover, the radiographic assessment of JSN can be influenced by the positioning of the knee and the X-ray beam [6]. The US FDA has an accelerated pathway for the approval of drugs used in serious diseases and accepts surrogate endpoints as the predictor of clinical benefit [20]. Recently, the FDA formally recognized OA as a serious disease and is open to considering suitable surrogate measures of appropriate clinical outcomes, such as imaging or biochemical markers associated with cartilage loss, as primary outcomes for clinical trials testing DMOADs [6]. Compared to radiography, magnetic resonance imaging (MRI) has provided a more responsive and consistent approach to demonstrating changes in OA structural features such as quantitative measures of cartilage volume and thickness [6]. As a result, an increasing number of clinical trials are using MRI to measure structural progression in OA. Recent long-term clinical trials, such as the FORWARD study, focusing on disease-modification in OA, show promising results [21,22]. However, the widespread use of MRI has cost and skilled human resources constraints; furthermore, the clinical significance of increased cartilage growth (cartilage volume and thickness) in the absence of clinically significant symptomatic relief is still a subject of debate [23].

## 3. Biochemical Markers

The biochemical molecules present in urine, synovial fluid, and blood as a result of the pathophysiological process indicate dynamic and measurable changes in the joint tissues reflecting joint remodelling and disease progression [23]. Unlike imaging biomarkers, biochemical markers provide a unique reflection of disease activity rather than current disease status [24]. Amid the ongoing research in this area, biomarkers such as C-telopeptide fragments of type II collagen (CTX-II) and serum cartilage oligomeric matrix proteins (COMP), along with few others, that represent catabolic changes have emerged as a promising candidate [25]. These biochemical markers provide insights into altered joint tissue homeostasis, joint inflammation, and altered subchondral bone remodelling in OA. However, despite much active research into this area, no single biochemical marker is satisfactorily well-recognized for the diagnosis or prognosis of OA and functions as a credible secondary or supportive endpoint outcome measure in clinical trials of DMOADs [25]. Nonetheless, from a health economic perspective, biochemical markers are slightly better positioned than imaging markers, which may help them cross the crucial translational barrier by providing an advantage in terms of cost per quality-adjusted life-year (QALY) gained [26].

## 4. OA Biomarkers and Personalised Medicine

Biomarkers are now increasingly used to characterize and subgroup (endotypes and phenotypes) patients with OA according to the BIPEDS classification. However, the results from these studies are not consistent in showing a sensitive marker that can predict joint pain, symptomatic OA, radiographic OA, or total joint replacement. However, clinical research in OA is focusing on exploring a personalized medicine approach using biomarkers—matching the right treatment with the right patient—to find an optimal management strategy to improve joint pain and change the disease trajectory [27]. Considering that OA is a heterogeneous disease with many endotypes and phenotypes, a personalized medicine strategy to identify the optimal population makes sense [28]. Some of these markers can play an important role in clinical trials to explore an individual-patient-based approach to test DMOADs. For example, our CurKOA trial tested *Curcuma longa* extract in knee OA patients with ultrasonography-defined effusion–synovitis (local inflammatory phenotype), and the ZAP2 trial assessed the effects of intravenous zoledronic acid in knee OA patients with subchondral bone marrow lesions (BML) detected by MRI (subchondral bone pathology phenotype) [29,30]. Similarly, encouraging results from a post hoc analysis of the CANTOS trial consisting of a population with elevated high sensitivity C-reactive protein (hsCRP) levels further support the exploration of the personalized medicine approach in OA [31].

## 5. Conclusions

In addition to the use as an outcome measure in clinical trials and as a measure to phenotype OA, biomarkers are essential as an outcome measure in population-based epidemiological studies [32]. Further, these markers can act as a surrogate measure of early OA, especially in young adult cohorts that help us to increase our knowledge of OA’s pathophysiology and risk factors [33]. Ultimately, these studies will inform risk factors of OA and targets for intervention and prevention strategies. These could have great potential for substantial cost savings through reductions in joint replacement surgery and improvements in the HRQoL in OA patients.
